# Is Guillain–Barre syndrome following chickenpox a parainfectious disease? A case report and literature review

**DOI:** 10.1186/s12883-023-03185-8

**Published:** 2023-03-30

**Authors:** Bademain Jean Fabrice Ido, Sidi Mahamoud Guebre, Emeline Agathe Carama, Alfred Anselme Dabilgou, Christian Napon

**Affiliations:** 1 Department of Neurology, University Hospital of Bogodogo, Ouagadougou, Burkina Faso; 2 Department of Neurology, University Hospital Yalgado Ouedraogo, Ouagadougou, Burkina Faso

**Keywords:** Polyradiculoneuropathy, Guillain-Barré syndrome, Varicella Zoster Virus, Africa

## Abstract

**Background:**

Polyradiculoneuropathy following infection with varicella zoster virus (VZV) is rare and most of the time, happens in the context of reactivation of latent VZV. We report a case of acute polyradiculoneuropathy following primary infection with VZV marked by atypical clinical features raising the hypothesis of a para-infectious disease.

**Case presentation:**

We describe a 43-years-old male who developed ataxia, dysphagia, dysphonia, and oculomotor disorders (vertical binocular diplopia and bilateral ptosis) followed by quadriplegia with areflexia which occurred 4 days later. The patient had a history of varicella that occurred 10 days before the onset of these symptoms. Nerve conduction study revealed features consistent with an acute motor-sensory axonal neuropathy (AMSAN). Anti-ganglioside antibodies were negative.

Based on clinical presentation and ancillary examination, we retain the Miller Fisher/Guillain-Barré overlap syndrome diagnosis.

The patient was treated with high doses of methylprednisolone but the evolution of the disease was nevertheless marked by a complete recovery six weeks after onset of symptoms.

**Conclusion:**

GBS following varicella is a rare but severe disease occurring most often in adults and marked by greater involvement of the cranial nerves. Its clinical features suggest that it is a para-infectious disease. Antiviral therapy has no effect on the course of the disease but its administration within the first 24 h after the onset of chickenpox in adults can prevent its occurrence.

## Background

Guillain-Barré Syndrome (GBS) is an acute autoimmune inflammatory demyelinating polyradiculoneuropathy, clinically characterized by progressive motor weakness, areflexia with albuminocytologic dissociation on cerebrospinal fluid (CSF) study [1].

In most cases, GBS is a post-infectious disease, and the most reported pathogens are Campylobacter Jejuni, Cytomegalovirus, Epstein-Barr Virus, Measles Virus, Influenza A Virus, Mycoplasma Pneumonia, Enterovirus D68, and Zika Virus [1, 2]. The type of preceding infection may influence the clinical phenotype, course and outcome of the disease [3].

In the literature, Varicella zoster virus (VZV) is rarely cited as a trigger for GBS and it is most of the time in the context of reactivation disease from latent VZV [4]. Clinically, GBS following VZV infection is characterized by sensory-motor impairment with a greater involvement of cranial nerves [3].

The following is a report of a 43-year-old man hospitalized in April 2022 in the neurology department of Bogodogo University Hospital (Burkina-Faso) for a polyradiculoneuropathy following primary infection with VZV marked by atypical clinical features. Through this clinical case and a review of the literature we discuss the pathophysiology underlying this particular presentation, and the possible therapeutic implications.

## Case presentation

It was a 43-year-old male working at a gas station as an attendant. He had a history of chickenpox which occurred on 06 April 2022, and was treated with paracetamol (1 g three times daily in case of fever) and skin antiseptic (Chlorhexidine gluconate). He was admitted to Bogodogo University Hospital's Neurology Department (Burkina-Faso) on 20 April 2022, for weakness of all four limbs, associated with dysphagia, dysphonia, and oculomotor disorders.

The onset of symptoms on 16 April 2022, was characterized by balance and gait disorders associated with a sensation of dizziness upon awakening. Two days later, the evolution was marked by swallowing disorders, a change in the timbre of the voice, and binocular diplopia. On the 5th day, the patient noted a weakness in the four limbs with tingling in the hands and feet.

At the admission, our examination found a normal consciousness, a proximal–distal weakness of the four limbs (2/5 proximally and distally according to MRC scale), a diffuse osteotendinous areflexia, involvement of the cranial nerves III, IX, and X, marked by vertical ophthalmoparesis (more noticeable on the left) and bilateral ptosis (III), dysphonia and abolition of nauseated reflex (IX) and dysphagia (X) without the respiratory disorder. The examination of gait, balance, and coordination was impossible due to the limb's weakness. No sphincter disturbance was noted. A dermatological analysis revealed chickenpox scar lesions that predominated on the face and the thorax. The patient had no history of chickenpox in childhood and was not vaccinated against chickenpox.

**Fig. 1:**
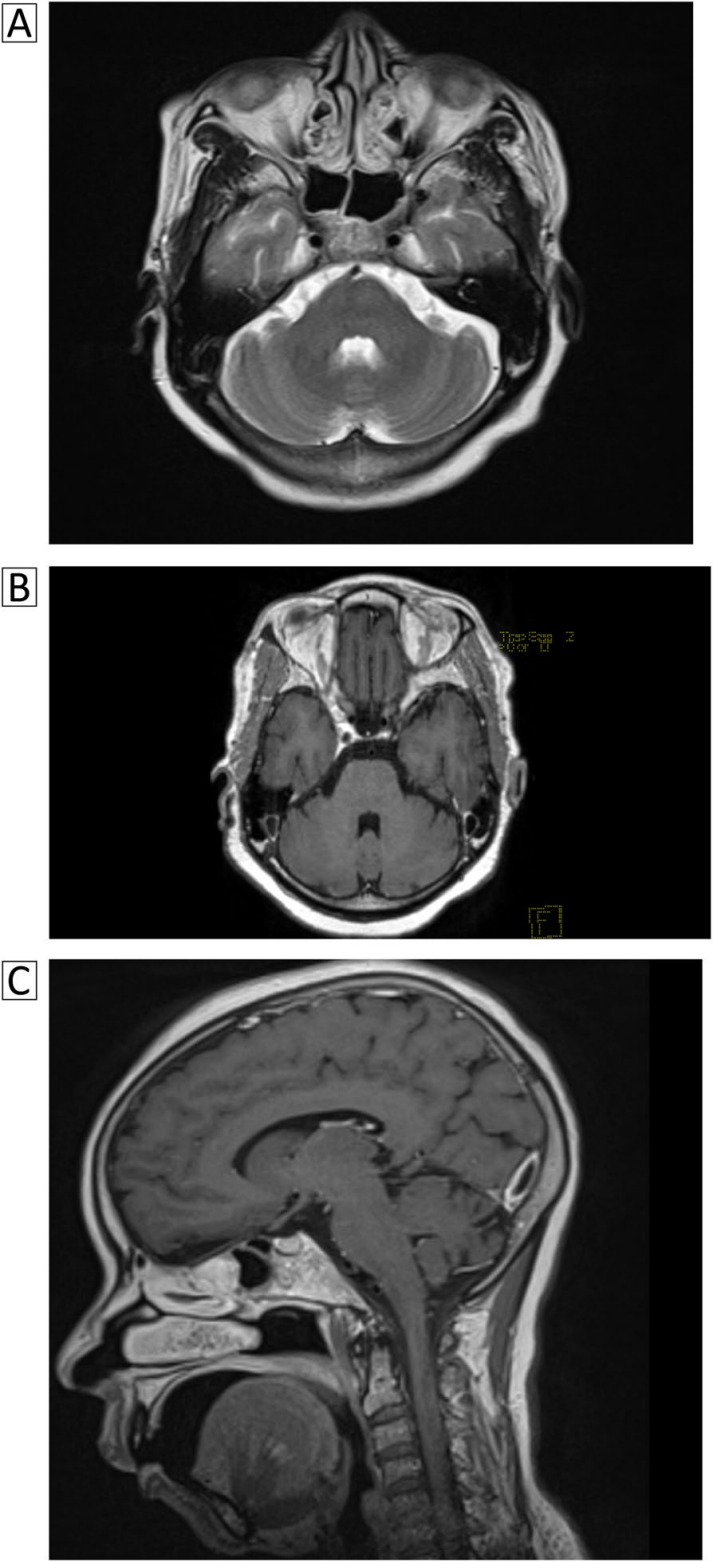
MR imaging findings. MR imaging of the cerebellum and the brainstem did not reveal findings suggestive of rhombencephalitis. **A**. Axial T2-weighted sequence.** B**. Axial T1-weighted with gadolinium sequence. **C**. Sagittal T1-weighted with gadolinium sequence

At this stage, GBS, Miller-fisher syndrome (MFS) and acute rhombencephalitis were considered. The usual biological assessment (blood count, C-reactive protein, complete blood ionogram, renal and hepatic function) came back normal. CSF analysis performed seven days after the onset of symptoms found normal proteinorachia and glycorrhachia without cells and germ. The search for anti-ganglioside antibodies (GM1, GM2, GM3, GM4, GD1a, GD1b, GD2 GD3, GT1a, GT1b, GQ1b) was negative for both IgM and IgG. VZV serology was positive for IgM and IgG, VZV testing in the CSF was not done (no technical facilities). The electroneuromyography (ENMG) performed 15 days after the onset of symptoms found increased latencies, reduced Compound Muscle Action Potentials (CMAP) amplitude, and reduced motor conduction velocities in all four limbs. Sensory nerves conduction study noted a reduction of Sensory Nerve Action Potentials (SNAP) amplitude more marked in lower limbs, with normal sensory conduction velocities (Tables 1 and 2). The Brain MRI was normal (Fig. 1A, B, C).

**Table 1:** Motor nerve conduction parameters

	**Right median**	**Left median**	**Right cubital**	**Left cubital**	**Right fibular**	**Left fibular**
Motor conduction velocity (MCV) (m/s)	40	38	42	41	-	-
Distal latencies (ms)	6.5	5.38	6.3	6.1	11.25	10.81
F wave latencies (ms)	38	36.3	37.5	37.3	Absent	Absent
Compound muscle action potential (CMAP) (mV)	2.1/1/0.8	1.5/1.1/0.9	2.3/1.4/1.4	2/1.4/1.3	0.3/0/0	0.7/0/0

**Table 2:** Sensitive nerve conduction parameters

	**Right median**	**Left median**	**Right cubital**	**Left cubital**	**Right sural**	**Left sural**
Sensitive conduction velocity (SCV) (m/s)	45	46	46	48	42	40
Sensory nerve actions potentials (SNAP) (μV)	12,4	13.7	8.9	7.3	4.8	4.3

Based on the normality of the Brain MRI with gadolinium, we eliminate the hypothesis of rhombencephalitis. However, concerning GBS and MFS, the clinical presentation and ancillary exams, did not allow us to eliminate any of them. Thus, we retained the Miller Fisher/Guillain-Barré overlap syndrome diagnosis.

The patient received corticosteroid therapy at a dose of 500 mg/day of methylprednisolone for five days.

The evolution was positive with the total regression of dysphagia, dysphonia, and ophthalmoplegia with an improvement in motricity two weeks after the onset of symptoms (3/5 proximally and 4/5 distally according to MRC scale). We then transferred the patient to the rehabilitation unit. The patient, seen four weeks after admission to the rehabilitation unit, had recovered his motricity entirely, the examination of the cranial nerves was normal, and he was no longer presenting any complaints.

## Discussion and conclusion

The patient presented clinically and electrophysiologically with an overlapping MFS and GBS syndrome. In the literature, several overlapping syndromes between GBS and MFS have been described [2, 5], and cranial nerves involvement seems to be the common point between these forms [5, 6].

Our patient had chickenpox a few days before his episode. Although rarely cited as a trigger for GBS spectrum diseases, studies have clearly shown a link between VZV infection and the occurrence of these diseases [7, 8]. Islam et al. [3] found in their literature review that cases of GBS occurring after chicken pox are mainly characterized by sensory-motor impairment, with cranial nerves involvement in 62% of cases could be. This is consistent with the clinical presentation of our study patient.

This greater involvement of the cranial nerves could find its explanation in the fact that VZV directly infects the spinal and geniculate ganglia a few weeks after the primary infection, where it remains latent for the rest of life [9]. Indeed, several arguments suggest that post-varicella polyradiculoneuropathy could be a para-infectious disease secondary to either the infectious process itself by direct attack by the virus [10], or an excessive and aberrant inflammatory immune response to the presence of the virus as described for SARS-CoV-2 [11]. Among these arguments, first, we have the short latency (less than 14 days) between the rash and the onset of neurological symptoms noted by most of the authors [3, 4, 12, 13]. Then, it was shown that VZV, which is known to infect T lymphocytes, remodels them. This could cause direct damage to the infected tissues, in particular the peripheral nerves [14, 15]. Moreover, the anti-ganglioside antibodies usually found in classic (post-infectious) cases of GBS are most often absent [3, 4, 12].

The absence of the virus in the CSF in most of the cases published and the ineffectiveness of antiviral therapy administered in some cases [12], seem to be more in favor of the excessive inflammatory immune reaction than a direct aggression by the virus. This virulence of the inflammatory immune reaction during the primary infection with VZV occurs particularly in adult and immunocompromised subjects [16]. Indeed, the majority of cases described in the literature were adult subjects. These patients had either not received antiviral treatment, or had received it several days after the onset of the rash. Unlike children, in whom it is a mild disease generally requiring only symptomatic treatment, in adults, chickenpox can be serious and an antiviral must be administered as quickly as possible to reduce viremia and serum antibodies to VZV [16]. The administration of acyclovir is recommended in the 24 h following the onset of the rash at a dose of 20 mg/kg (maximum dose of 800 mg) 4 times a day for 5 days [16].

The vaccination of adults who escaped chickenpox in childhood is also an effective way to prevent the occurrence of these severe forms. Although it is very rarely offered in several countries as chickenpox is perceived as a mild disease.

The indicated treatment in post-varicella GBS is either intravenous immunoglobulins (IVIg) or plasma exchange [3, 12]. Our patient received high doses of methylprednisolone due to the unavailability of plasma exchanges in the country and the very high cost of immunoglobulins, making them inaccessible without health insurance. Despite this unsuitable treatment [17, 18], and the fact that the disease is described as having a poor prognosis [13], the evolution was nevertheless marked in our case by a complete recovery of the patient 4 weeks after the onset of symptoms. A possible explanation would be the good prognosis of the MFS variant of GBS [19, 20].

In conclusion, GBS following varicella is a rare but severe disease occurring most often in adults and marked by greater involvement of the cranial nerves. The very short latency between the appearance of the rash and the occurrence of the polyradiculoneuropathy suggests that it is a para-infectious disease. Antiviral therapy has no effect on the course of the disease but its administration within the first 24 h after the onset of chickenpox in adults can prevent its occurrence.

## Data Availability

The data generated for this study are accessible upon request to the first author, Bademain Jean Fabrice Ido (idojfabrice@yahoo.fr).

## Abbreviations

GBS: Guillain-Barré syndrome
MFS: Miller-Fisher syndrome
VZV: Varicella Zoster Virus
CSF: Cerebrospinal fluid
ENMG: Electroneuromyography
CAMP: Compound Muscle Action Potentials
SNAP: Sensory Nerve Action Potentials
